# Disintegration of simulated drinking water biofilms with arrays of microchannel plasma jets

**DOI:** 10.1038/s41522-018-0063-4

**Published:** 2018-10-18

**Authors:** Peter P. Sun, Elbashir M. Araud, Conghui Huang, Yun Shen, Guillermo L. Monroy, Shengyun Zhong, Zikang Tong, Stephen A. Boppart, J. Gary Eden, Thanh H. Nguyen

**Affiliations:** 1 Department of Civil and Environmental Engineering, University of Illinois, Urbana, IL 61801 USA; 2 Department of Electrical and Computer Engineering, University of Illinois, Urbana, IL 61801 USA; 3 Department of Bioengineering, University of Illinois, Urbana, IL 61801 USA; 4 Present Address: Department of Civil and Environmental Engineering, University of Michigan, Ann Arbor, MI 48109 USA

## Abstract

Biofilms exist and thrive within drinking water distribution networks, and can present human health concerns. Exposure of simulated drinking water biofilms, grown from groundwater, to a 9 × 9 array of microchannel plasma jets has the effect of severely eroding the biofilm and deactivating the organisms they harbor. *In-situ* measurements of biofilm structure and thickness with an optical coherence tomography (OCT) system show the biofilm thickness to fall from 122 ± 17 µm to 55 ± 13 µm after 15 min. of exposure of the biofilm to the microplasma column array, when the plasmas are dissipating a power density of 58 W/cm^2^. All biofilms investigated vanish with 20 min. of exposure. Confocal laser scanning microscopy (CLSM) demonstrates that the number of living cells in the biofilms declines by more than 93% with 15 min. of biofilm exposure to the plasma arrays. Concentrations of several oxygen-bearing species, generated by the plasma array, were found to be 0.4–21 nM/s for the hydroxyl radical (OH), 85–396 nM/s for the ^1^O_2_ excited molecule, 98–280 µM for H_2_O_2_, and 24–42 µM for O_3_ when the power density delivered to the array was varied between 3.6 W/cm^2^ and 79 W/cm^2^. The data presented here demonstrate the potential of microplasma arrays as a tool for controlling, through non-thermal disruption and removal, mixed-species biofilms prevalent in commercial and residential water systems.

## Drinking water: fighting biofilms with plasma jets

Biofilms in drinking water premise plumbing systems can be disrupted and their microorganisms deactivated by exposure to jets of ionized gas known as plasma. Researchers at the University of Illinois, USA, led by Thanh (Helen) Nguyen and J. Gary Eden, explored the potential of low temperature plasma jets in disrupting & removing drinking water biofilms. The plasma was directed through fabricated microchannels and onto samples that the simulated biofilms. The interaction of the plasma with air and water generated reactive chemical species and ultraviolet radiation that disrupted the biofilms and deactivated the microorganisms within them. The biofilms studied vanished within 20 min. of plasma exposure. Plasma jets offer an inexpensive, low temperature and chlorine-free method for combating harmful biofilms in drinking water premise plumbing systems.

## Introduction

Biofilms are ubiquitous in municipal and residential drinking water distribution systems, and they present significant human health concerns because of their ability to harbor pathogens^1^. It has long been known that biofilms are able to serve as pathogen reservoirs by supplying nutrients^2–6^ and shielding pathogens from disinfectants^7,8^. In particular, biofilms have been shown to capture and accumulate planktonic pathogens and eventually release these species into air or a water flow stream by the detachment of biofilm material^1^. As one example, a recent University of Virginia study^9^ detected, in the plumbing of several sinks, the vertical propagation of biofilm from the trap to the strainer and the subsequent dispersal of *E. coli* in the form of an aerosol. Although residual disinfectants in drinking water are mandated by the U.S. Environmental Protection Agency (EPA) for microorganism control in drinking water distribution systems, biofilms persist despite exposure to disinfectants because the extracellular polymeric substance produced by biofilms consumes the disinfectant, thereby hindering or preventing disinfectant permeation^10,11^.

Low temperature plasma generated in a gas flow stream is able to efficiently produce molecular radicals capable of deactivating pathogens^12,13^. Both the fungi and bacteria often found in tooth canals^14,15^ or implanted medical devices^12,13,16^, for example, have been successfully treated with plasma. While the efficacy of low temperature plasma in disrupting and removing single species biofilms, and deactivating the pathogens they harbor, has been reported^17–20^, little is known of the impact of plasma on the structure of the complex multi-species biofilms of municipal water systems.

We report here the disruption and erosion of biofilms, grown under simulated premise plumbing conditions, with an array of microplasma jets generated in microchannel/electrode structures fabricated by 3D printing. An extensive suite of diagnostics examined the deformation and removal of the biofilms during plasma treatment, as well as the reactive oxygen species produced by the interaction of the helium (He) plasma micro-columns with room air. Specifically, optical coherence tomography (OCT) shows that the thickness of simulated water biofilms falls from a mean value of 122 ± 17 µm to 55 ± 13 µm after 15 min. of exposure to a microplasma array operating at a modest power density (58 W/cm^2^). With 20 min. of exposure at the same power density, the biofilms vanish. Increased biofilm removal rates are readily available with larger dissipated power densities (up to 78 W/cm^2^ in the present experiments). Furthermore, analysis of treated biofilms by confocal laser scanning microscopy (CLSM) reveals that the number of living cells remaining in the biofilm (following 15 min. of exposure to the plasma) is reduced by 93% with respect to the control. No living cells are detected after 20 min. of exposure of samples to the microplasma array. Concentrations of the hydroxyl radical (OH), singlet oxygen (^1^O_2_), hydrogen peroxide (H_2_O_2_), and ozone (O_3_) produced by the microplasmas were measured by liquid chromatography or colorimetry (in the case of hydrogen peroxide), and it is these species that appear to be primarily responsible for the destruction of the biofilms and the deactivation of the pathogens they contain. Aside from the sensitivity of the present experiments (nanomolar per second) in measuring the generation rates for hydroxyl radicals and singlet oxygen produced by the microplasmas, the primary significance of the results reported here is the demonstration of an effective tool with which the growth of biofilms in drinking water distribution networks can be mitigated. Furthermore, the insertion of microplasma arrays of cylindrical geometry into commercial or residential plumbing systems, in a fashion similar to that of conventional plumbing snakes, appears to be feasible. Such a capability will permit selective intervention into building plumbing for drinking water at locations that have resisted previous attempts at disinfection.

The approach proposed here for the control and removal of biofilms represents a significant departure from water system treatments commonly used in the U.S. which have typically involved chlorination. Through the choice of the feedstock gas and the microplasma jet characteristics (electron temperature, in particular), one is now able to specify the predominant radicals and molecular excited species generated by the plasma and its interaction with ambient air. Precisely tailoring the plasma chemistry at the point of use and matching particular plasma-generated species with specific biofilms is now possible. The *in situ* application of low temperature plasma jets to biofilm control and removal has no precedent, and yet offers targeted treatment (localized and tailored to specific biofilm compositions or structures) of residential and commercial water systems.

## Results

### **Design and performance of 9** **×** **9 microchannel plasma jet arrays**

**Fig. 1:**
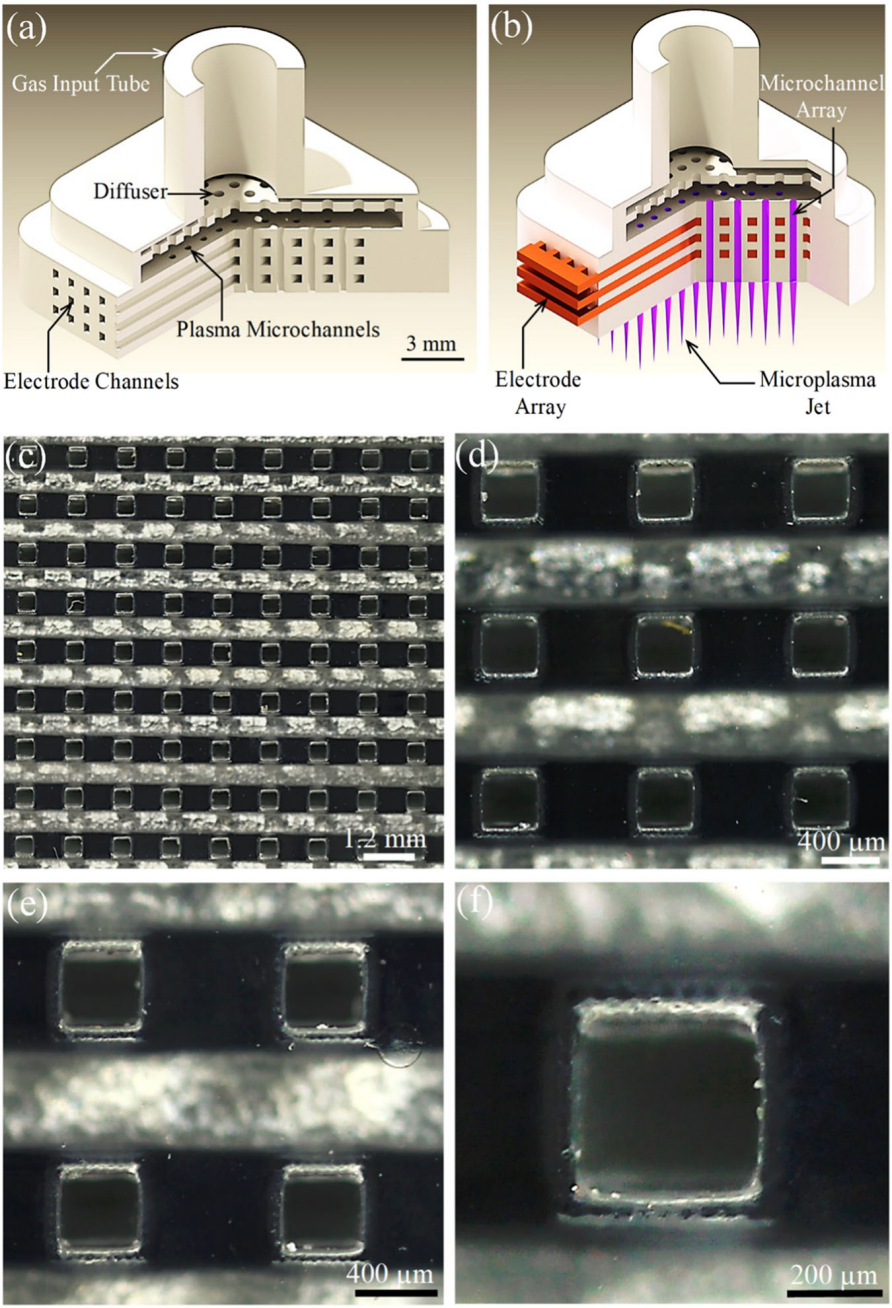
Diagrams (in cutaway view) of the 3D-printed microplasma jet array devices: **a** monolithic structure prior to the insertion of electrodes; **b** illustration of the installed electrode arrays (in red) and the array in operation, producing multiple plasma jets (in blue). Feedstock gas (He in the present experiments) is injected by means of the tube at top, and the applied electric field is oriented parallel to the direction of gas flow: (see through f); Optical micrographs of a 9 × 9 array of square cross-section microchannels, shown at increasing levels of magnification: **c** the full 9 × 9 array; **d** a 3 × 3 section of the full 81 microchannel array; **e** a 2 × 2 array segment (the lower of the two horizontal lines at the bottom of all four channels is a spurious optical reflection); **f** a single microchannel, showing the spatial resolution of the 3D printing process

Two diagrams of the structure adopted for the microchannel plasma jet array device are presented in Fig. 1a, b. Both are cutaway views of the monolithic design, and panel (a) of the figure illustrates the structure prior to the insertion of the electrode arrays. After entering the 4 mm i.d. input tube (at top, Fig. 1a), the feedstock gas (He, 99.99%) encounters a microchannel diffuser and a shallow plenum region which serve the purpose of minimizing turbulence^21^ and directing gas equally to each of the square cross-section microchannels. This entire structure was fabricated in a transparent polymer by 3D printing. The electrode arrays have a multi-finger design and are machined in copper. When the electrodes are installed (Fig. 1b), this device generates a 9 × 9 array of microplasma columns that emerge from the lower face of the device, producing visible plumes extending approximately 1 mm into room air. It must be emphasized that the array design of Fig. 1a, b allows for the electrode arrays to also be inserted so as to be oriented in a plane parallel to the gas flow. However, for the experiments described here, the electrodes are situated as illustrated in Fig. 1b.

Optical micrographs of the electrode/microchannel structure of the array are given in Fig. 1c–f. Each channel has a 400 × 400 µm^2^ square cross-section, and the images show one or more microchannels and their associated electrodes at successively greater levels of magnification. A full 9 × 9 array of microchannels, having an overall area of 125.4 mm^2^ (1.25 cm^2^), is presented in Fig. 1c whereas only a single microchannel is shown in panel (f). The pitch (center-to-center spacing) between adjacent channels is 1.2 mm along both the horizontal and vertical axes of the two-dimensional array which defines the areal packing density of the array as 88 channels/cm^2^. As noted earlier, all of the arrays in these experiments were constructed with a 3D printing tool having a spatial resolution of 50 µm. Panel (f) of Fig. 1 shows that, aside from a slight “bowing” of the sidewalls of the channel, this limitation in the tool resolution has no significant adverse consequences.

**Fig. 2:**
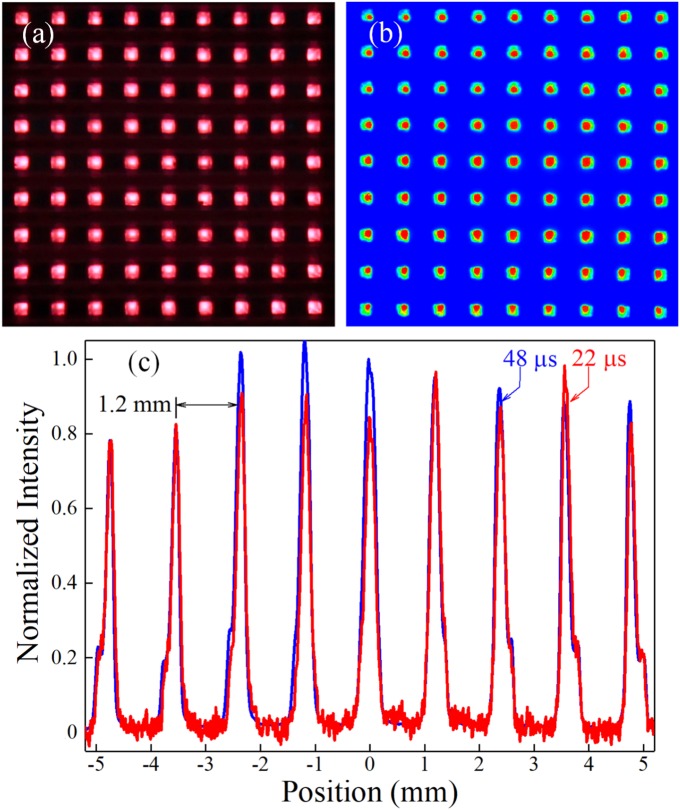
End-on optical images and lineouts illustrating the uniformity of the microplasma columns emerging from the array device: **a** Visible emission produced from each of the 81 channels in the array when He is the feedstock gas; **b** false color image of the emission, and **c** lineouts of the array emission recorded 22 µs (red) and 48 µs (blue) following the zero-crossing of the positive half-cycle of the sinusoidal voltage waveform. The microchannels represented in **c** are those forming the central column in the arrays of **a** and **b**. All images were recorded with a 20 kHz sinusoidal voltage having an RMS value of 1.2 kV

Figure 2 presents time-integrated images of the visible emission produced by a 9 × 9 array of He plasma jets when the He mass flow rate was fixed at 0.25 L/minute/microchannel. Figure 2a is an end-on view of the fluorescence generated when the array is driven by a 20 kHz sinusoidal voltage waveform (1.2 kV RMS), and it is clear that the jets are equally spaced along the two orthogonal coordinates of the array. The result of integrating over a 50 µs window (a full cycle of the driving voltage), the intensified charge-coupled device (ICCD) camera image of Fig. 2a demonstrates that spatially uniform glow plasmas are produced within the square microchannels, but the most intense emission emanates from a cylindrical region having a diameter of 200–300 µm. Images similar to that of Fig. 2a were recorded for power densities of 3.6–79 W/cm^2^ dissipated by the array. Panel (b) is a false color image of the intensity map (derived from images such as that of Fig. 2a) acquired for a power density of 58 W/cm^2^, and demonstrates the uniformity of the peak emission over the entire array. Two intensity lineouts of the central column of microchannel plasmas (Fig. 2c) show that the difference between the integrated intensity produced by the interior channels differs by less than 25% from that emanating from the channels at the perimeter of the array. The lineouts of panel (c) were derived from images recorded over a 500 ns interval centered at *t* = 22 µs (red) or *t* = 48 µs (blue) after the zero-crossing for the positive half-cycle of the 20 kHz voltage waveform.

**Fig. 3:**
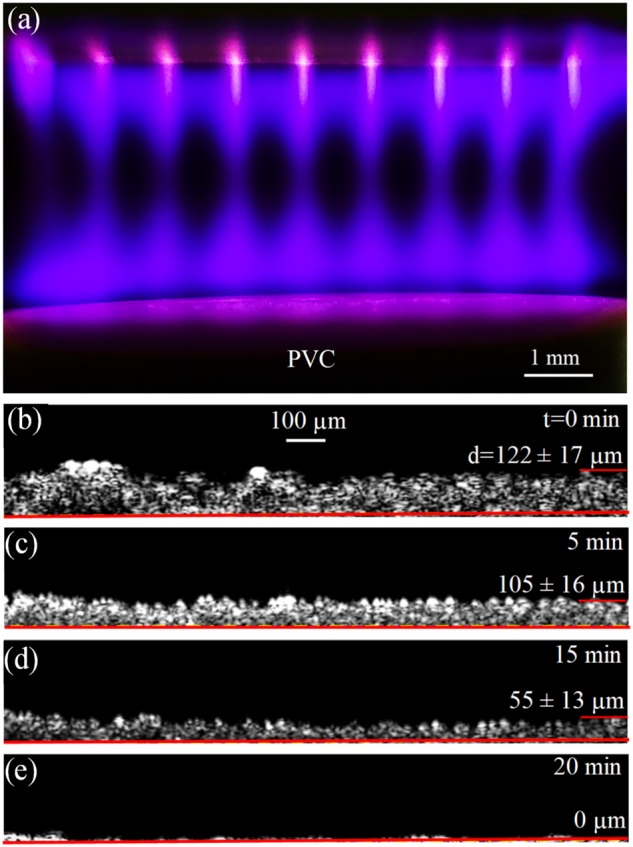
**a** Optical image of a column of microplasma jets emerging from the array device (top) and impinging on a PVC biofilm coupon which was located 4.0 mm from the exit face of the array. The He gas flow was maintained at 0.25 L/min/microchannel and the red, horizontal region indicates the surface of the coupon; **b**–**e** (in cross-section) of a biofilm, prior to and following exposure to the He microplasma arrays for three time periods (*t*): 5, 15, and 20 min. For *t* = 20 min., no biofilm is detectable to within experimental uncertainty. The solid red line denotes the boundary between the biofilm and the surface of the PVC coupon

Figure 3a is a side view of the optical emission recorded with a Cannon camera (Canon 5D Mark III) during the irradiation (in room air) of one of the biofilm coupons. This image illustrates vividly the interaction of the He plasma with air to produce molecular radicals, ions, and excited species. Once the microplasmas emerge into room air, the red fluorescence of the He plasma columns is transformed into blue emission as a result of the Penning ionization of the nitrogen molecule by the He(2^3^*S*) metastable species. The B-X electronic transition of the $N_2^ +$ ion is responsible for the violet/blue fluorescence evident above the biofilm coupon. Note that the breadth of the $N_2^ +$ blue emission profiles is greater than that of the He microplasmas, owing to the diffusion of He metastables into the cold surrounding gas.

### **OCT and CLSM images for treated and untreated biofilms: biofilm erosion and cell deactivation**

OCT images of the biofilms were recorded *in situ*, both prior to exposure to the He microplasma array and at several points during the treatment process. Representative results are shown in cross-section in Fig. 3b–e where the first panel (Fig. 3b) is a profile of the film prior to plasma treatment. Figures 3c-e illustrate the impact on the structure and average thickness (*d*) of the film to exposure to the plasma array for time periods (*t*) of 5, 15, and 20 min. Throughout these tests, the electrical power delivered to the array was fixed at 3.4 W. In each biofilm image of Fig. 3, the horizontal line indicates the boundary between the biofilm and the surface of the PVC coupon. Note that the biofilm thickness decreases from an initial value of 122 ± 17 µm (where the uncertainty represents one standard deviation) to 55 ± 13 µm after 15 min. of exposure to the plasma array. This represents a decline in thickness of 55% and a linearized biofilm erosion rate of 4.5 µm/min. After 20 min. of plasma treatment, no biofilm is detectable to within experimental uncertainty. Consequently, the temporal rate of biofilm removal by the microplasma jets is decidedly nonlinear which is attributable to the time required for the radicals, and excited species, produced by the microplasma columns to diffuse through one atmosphere of air and reach the coupon.

**Fig. 4:**
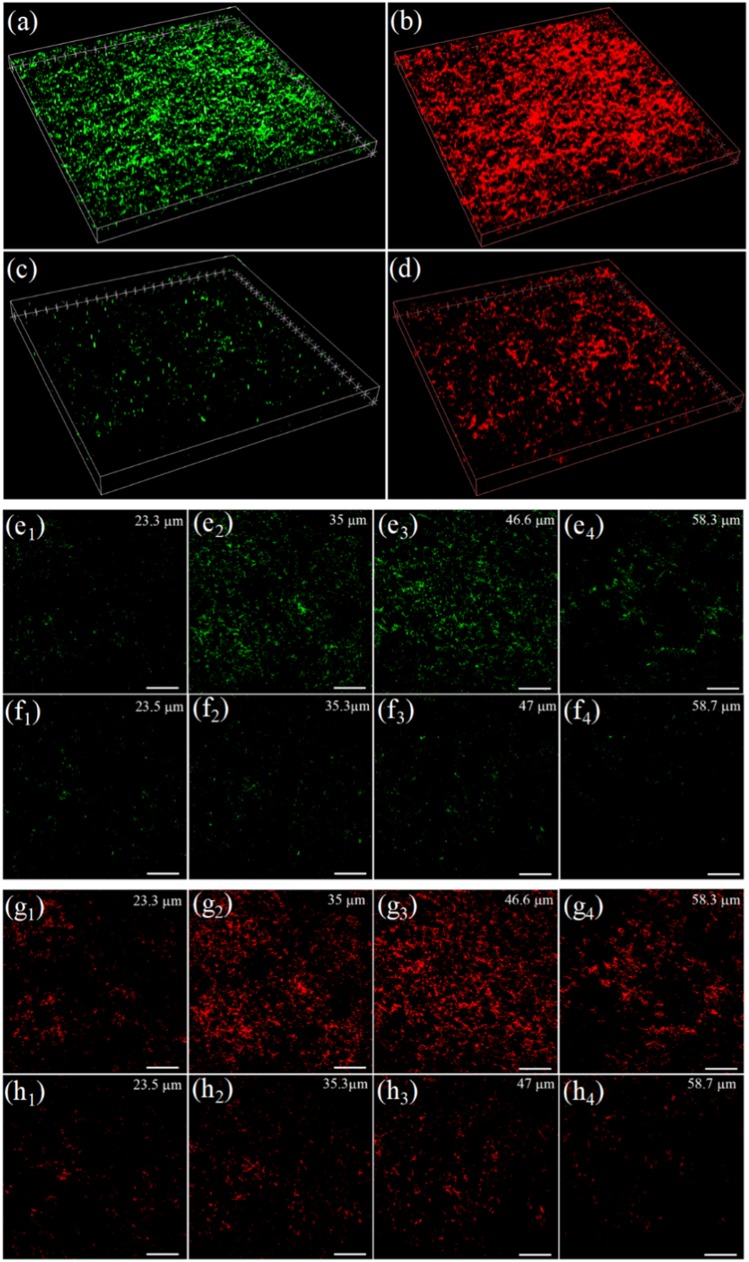

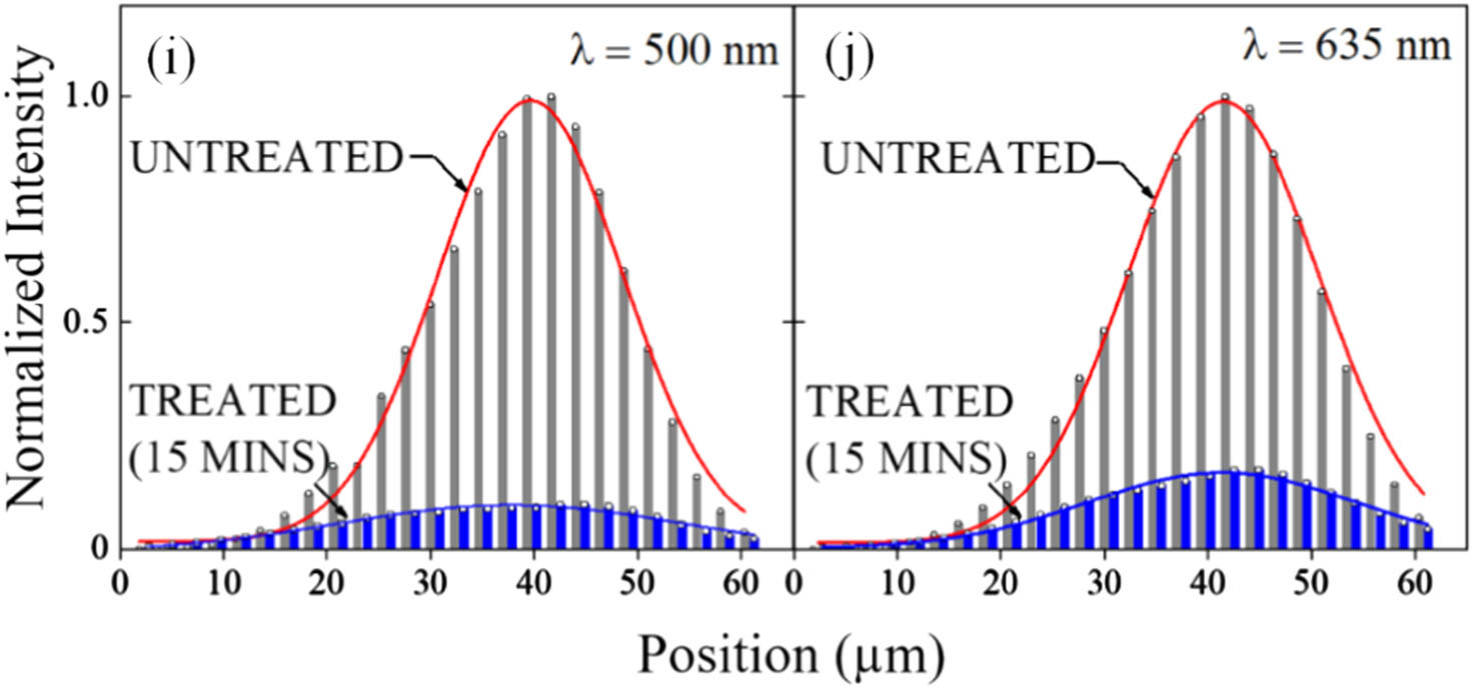
CLSM images of the biofilm of Fig. 3, recorded prior to and after exposure of the film to the microplasma array: **a**, **b** 3D images of the biofilm at *t* = 0 (immediately prior to microplasma treatment), indicating the distribution of living cells (panel **a**, SYTO 9 fluorophore, *λ* = 500 nm) and deactivated cells (**b**, propidium red fluorophore, *λ* = 635 nm); **c**, **d** CLSM images corresponding to those of **a** and **b**, respectively, but recorded after 15 min. of treatment of the biofilm with low temperature plasma; **e**–**h** Series of CLSM images recorded for SYTO 9 (green, *λ* = 500 nm) staining, at several values of depth into a biofilm (**e**_1_–**e**_4_, **f**_1_–**f**_4_). The row of images at top (**e**_1_–**e**_4_) were obtained prior to treatment of the biofilm with plasma, while those in the lower row (**f**_1_–**f**_4_) were acquired after 15 min. of exposure to the microplasma array. The white horizontal bars in each panel represent a length of 200 µm; **g**_1_–**g**_4_, **h**_1_–**h**_4_: Images corresponding to those of Fig. 4 **e**_1_–**e**_4_ and **f**_1_−**f**_4_ but recorded by mapping the fluorescence from propidium red (*λ* = 635 nm). Again, the horizontal bars represent a length of 200 µm; **i**, **j**: Summary of the green (SYTO 9, *λ* = 500 nm, panels **e** and **f**) and red (propidium red, *λ* = 635 nm, panels **g** and **h**) fluorescence depth profiles, measured for biofilms that were untreated (light gray bars) and those treated by exposure to the microplasma jet array for 15 min. (dark blue bars). The red curves represent the fitting of a Gaussian to the data

Images similar to, and in agreement with, those of Fig. 3 but displayed in three dimensions by confocal laser microscopy (CLSM) are presented in Fig. 4. Untreated biofilms, as well as those exposed to plasma treatment for 15 min., were stained and subsequently probed by CLSM. Panels (a) and (b) of Fig. 4 illustrate the spatial distribution of living and deactivated cells, respectively, existing in one film at *t* = 0, immediately prior to the exposure of the biofilm to microplasma. The green fluorescence (*λ* = 500 nm) from SYTO 9 in panel (a) denotes those cells with undamaged membranes, whereas the propidium red fluorescence (*λ* = 635 nm) indicates those cells that are deactivated because of ruptured or damaged membranes. After 15 min. of exposure of this same biofilm to the microplasma array, virtually all of the living cells have been deactivated, as evidenced by the pronounced decrease (relative to that of Fig. 4a) in green fluorescence in the SYTO 9 scan of Fig. 4c. Similarly, the rapid reduction in the biomass of the film is reflected by the propidium red image of Fig. 4d which shows a dramatic decline in 635 nm fluorescence. Again, it must be emphasized that before 20 min. of exposure was reached, all of the biofilms investigated vanished.

Depth-resolved studies of the green (SYTO 9) and red (propidium red) photoemission generated by CLSM were conducted for both untreated biofilms and those exposed for 15 min. to microplasmas and the species they produce in air. Figures 4e–h provide an overview of the images recorded in these experiments. Two-dimensional maps of the SYTO 9 (green) fluorescence are presented in Fig. 4e, f where the upper row of images (e_1_–e_4_) are those for the biofilm prior to treatment, whereas the bottom row (f_1_−f_4_) is associated with a film subjected to 15 min. of exposure. For both Fig. 4e, f, images recorded at depths of ~23, 35, 47, and 58 µm are presented. Images corresponding to those of Fig. 4e, f but obtained by recording the spatially resolved emission from propidium red are given in Fig. 4g, h. Figure 4i, j summarize the results of these experiments by illustrating the dependence of the spatially integrated SYTO 9 (or propidium red) fluorescence on depth into a biofilm. The light gray bars, derived from Figs. 4e–h by ImageJ and COMSTAT analysis (*cf*. Methods), indicate the variation with depth of the integrated, normalized fluorescence intensity for the untreated biofilms, and the blue bars represent treated biofilms. On the basis of these data, one concludes that green channel (SYTO 9) fluorescence decreases by approximately 90% when the biofilms are exposed to plasma for 15 min. Similarly, the red fluorescence (Fig. 4j) declines by 80% during that same time period because of the rapid erosion of the biofilm by the plasma array.

After 20 min. of biofilm exposure to the plasma array, no fluorescence—red or green—is detectable, a result consistent with Fig. 3e. Furthermore, these results have been confirmed by scanning electron microscopy (SEM) and energy dispersive spectroscopy (EDS) (Fig. 1S). The former shows no cells remaining at *t* = 20 min., and EDS reveals that plasma treatment reduces carbon content in the film from 18% (pre-treatment) to ~3% (at ≤ 20 min.). The factor of six decline in carbon content and the absence of any cells underscore the conclusion that the biofilm is removed by 20 min. of exposure. Dispersed biofilm fragments were collected and stained following the protocol of the LIVE/DEAD BacLight™ Bacterial Viability Kit. The images recorded by confocal laser scanning microscopy show no fluorescence (green or red), as is evident in Fig. 2S. In addition, several post-exposure samples observed by SEM showed no cells whatsoever (Fig. 3S).

### **Production of OH,**^**1**^**O**_**2**_**, H**_**2**_**O**_**2**_**, and O**_**3**_**by the microplasma jet arra**y

**Fig. 5:**
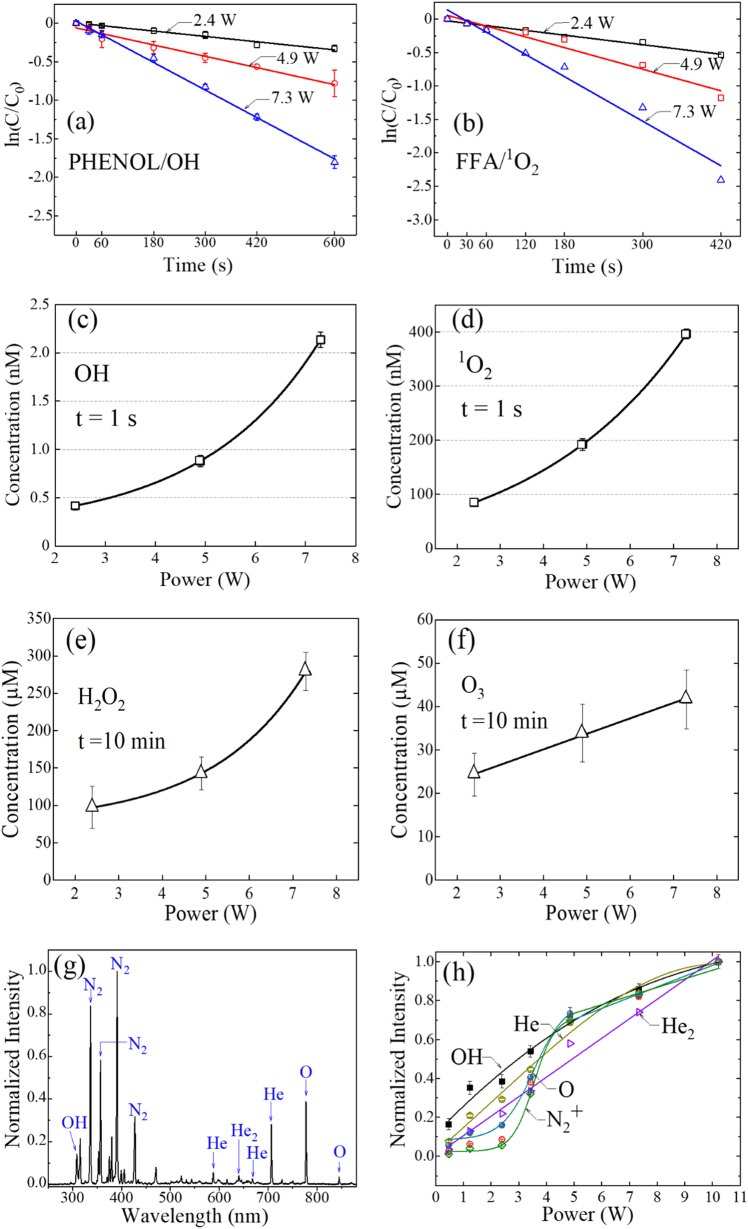
**a**–**d** Concentrations of OH, and ^1^O_2_ produced by a He microplasma jet array in phenol or FFA solutions: **a** Decay of the phenol concentration, C (relative to its initial value C_0_), for microplasma exposure times up to 600 s. Data are presented for several values of power delivered to the 9 × 9 array, and note that the ordinate is logarithmic; **b** Data similar to those of **a** but for FFA in a PBS solution; **c** Concentration of OH, calculated from the data of **a**, for three values of power dissipated by the plasma; **d** Dependence on microplasma array power of the ^1^O_2_ concentration (calculated from **b**). The results of panels **c** and **d** are those recorded after only one second of exposure of the phenol/PBS or FFA/PBS solutions to the plasma; **e**–**f**: Measurements of the concentrations of: H_2_O_2_, and O_3_ after 10 min. of plasma array exposure to PBS solutions. Data are given for three values of power (*P*) dissipated by the microplasma jet array; **g** Emission spectrum of the 9 × 9 microplasma jet array, recorded end-on over the 300–850 nm spectral interval. The He backing pressure for the jets was 785 Torr (300 K pressure), the driving voltage was a 20 kHz sinusoid, and the array emerged into room air; **h** Variation with peak driving voltage of the normalized fluorescence generated by several selected emitters. The oxygen atomic transition is the 777 nm line of panel **g**

Biofilm disintegration (Fig. 3b–e) is undoubtedly the result of reactive molecular species generated by the microplasma array. Several, in particular, are capable of rupturing critical chemical bonds in the biofilm. To indirectly test this hypothesis, we quantified the concentrations of selected reactive species produced in the liquid phase by the microplasma jet array by measuring the degradation kinetics of specific probe chemicals. Experiments reported here measured the absolute concentrations of OH, ^1^O_2_, H_2_O_2_, and O_3_, the first two of which were detected by phenol and furfuryl alcohol (FFA) solutions, respectively. Panel (a) of Fig. 5 presents data showing the decay, with increasing microplasma exposure time (*t*), of the concentration C of phenol (a diagnostic for OH), relative to an initial value C_0_. Results are given for three values of power dissipated by the microplasma jet array (*P* = 2.4, 4.9, and 7.3 W), and note that the ordinate is logarithmic. Measurements were recorded for array treatment times up to *t* = 600 s, and it is evident that the data exhibit a linear dependence on *t* which is to be expected if the interaction of microplasma - produced OH with phenol is a first-order process. Indeed, the solid lines in Fig. 5a represent the best fit of a pseudo-first order, rate constant kinetics model of the data and, for each value of dissipated power, the R^2^ value is ≥0.99. Therefore, these measurements are described well by the relation:

1 $${\mathrm{ln}}\left( {{\mathrm{C/C}}_{\mathrm{0}}} \right){\mathrm{ = }} - {\mathrm{A}} {{t}} {\mathrm{ + B}}$$

where A and B are constants. Furthermore, the rate of decay of the phenol concentration with plasma exposure time *t* rises with the level of power delivered to the plasma array. The rapid decline in the phenol concentration with *t* is attributed to the formation of OH radicals in the PBS solution, and panel (c) of Fig. 5 shows the dependence on array power of the OH radical concentration, calculated from Fig. 5a for *t* = 1 s. Note that the OH concentration is determined to be 0.4 nM when *P* = 2.4 W. Similarly, increasing the electrical power dissipated by the microjet array to 4.9 and 7.3 W raises the OH concentration (after only 1 s of plasma solution exposure) to 0.9 nM and 2.1 nM, respectively. Further support for the premise that the microplasma jet array produces substantial concentrations of hydroxyl radicals (OH) in the experiments of Fig. 5a is provided by introducing an antioxidant. Mannitol, for example, is known to be an OH scavenger and Fig. 4S demonstrates that the addition of mannitol (0.15 M) to the PBS/phenol solution almost completely offsets the plasma array production of OH.

Data corresponding to those of Fig. 5a, c are presented in panels (b) and (d), respectively, for FFA/PBS solutions exposed to the microplasma jet array. In this case, FFA is a probe for the ^1^O_2_ metastable molecule and the ^1^O_2_ concentrations are considerably higher than those for the OH radical, rising from 85 nM for 2.4 W of input power to the array, to 396 nM for 7.3 W of power dissipated. This result is not surprising because the loss of FFA in Fig. 5b is 91% after 7 min. of exposure to the microplasmas whereas the phenol reduction in panel (a) is 84% at *t* = 10 min. Recalling (*cf*. Methods) that the ^1^O_2_—FFA rate constant is two orders of magnitude smaller than that for the phenol-OH interaction, it is evident that the plasma jets interacting with room air generate ^1^O_2_ number densities considerably larger than those of the hydroxyl radical.

Figure 5e, f summarize the results for the H_2_O_2_ and O_3_ concentration measurements, respectively, in PBS solutions when the microplasma exposure time is *t* = 10 min. For H_2_O_2_, the measured concentration rises from 98 µM at 2.4 W of array power (P), to 280 µM for 7.3 W. Clearly, the hydrogen peroxide concentration rises linearly (overall) in this range of plasma array power but the preponderance of the increase occurs between *P* = 4.9 and 7.3 W. As shown in Fig. 5f, the O_3_ concentration is sublinear in the array power despite the linear appearance of the graph (owing to the suppression of zero on the abscissa), increasing from 24 µM at *P* = 2.4 W to 42 µM at 7.3 W.

For several of the molecular species of interest in Fig. 5a–f, the relative species concentration in solution is consistent with the observed dependence of the fluorescence emission intensity of a precursor excited atom, or molecule, in the gas phase. Panel (g) of Fig. 5 is a low resolution (∆*λ* ≈ 0.3 nm) spectrum of the spontaneous emission from the 9 × 9 microjet array, recorded over the 300–850 nm wavelength region. Dominated by the N_2_ (C → B) electronic transition that is responsible for molecular emission at 337.1, 357.7, and 380.5 nm, for example, this spectrum also shows contributions from the singly charged nitrogen ion ($N_2^ +$), atomic oxygen, and OH. Furthermore, the linear dependence of the OH (A → X) fluorescence intensity on the plasma array voltage, observed in Fig. 5h, is reflected in the variation of the OH and H_2_O_2_ concentrations (in solution) with the power dissipated by the microplasma array. Similarly, the nonlinear rise of the ^1^O_2_ concentration with increasing power delivered to the array (Fig. 5d) is to be expected because the population of the 3p ^5^P_2_ state of atomic oxygen (upper level of the 777 nm transition of Fig. 5g, h) also increases in a nonlinear manner with rising plasma voltage (and power). Owing to its internal energy of 10.7 eV, the O (3p ^5^P_2_) state is a potential precursor to ^1^O_2_ in both the gas and solution phases.

## Discussion

Exposure of simulated drinking water biofilms to the excited species, radicals, and ultraviolet radiation produced by a 9 × 9 array of microplasma jets is found to erode (and remove) the films, as well as deactivate the cells they contain. Measurements of biofilm thickness by OCT demonstrate that 122 µm thick films are removed entirely by 20 min. of exposure to the plasma array. Combined with the observation that the number of living cells in the biofilms falls by >93% with 15 min. of exposure (and reaches zero by *t* = 20 min.), these results indicate that arrays of low temperature plasma jets offer an inexpensive and nonthermal tool with which the growth of biofilms in drinking water distribution systems can be mitigated or eliminated.

A few additional comments regarding the applicability of this technology to residential or commercial water networks are warranted. Although the geometry of the microplasma array of Fig. 1 was chosen for experimental convenience, it is clearly not suitable for implementation in drinking water distribution systems. However, multiple configurations of microplasma jet arrays are currently available. One of these is the cylindrical geometry, described in U.S. Patent No. 8,957,572, in which microplasma jets propagate radially outwards from a central cylinder having a diameter that can be less than 6 mm (1/4”). Furthermore, the plasma microcolumns are produced in an azimuthally symmetric pattern and are spaced equally along the axial coordinate of the array. The length of such devices can be extended so as to increase the surface area being treated at any given time, thereby expediting the treatment process. Such cylindrical devices may be introduced to premise plumbing systems in a manner similar to that for conventional plumber “snakes” which typically include integrated CCD cameras. The simultaneous insertion of several snakes will accommodate portions of the plumbing system having copper or PVC pipe of differing diameters, thus decreasing treatment time.

**Fig. 6:**
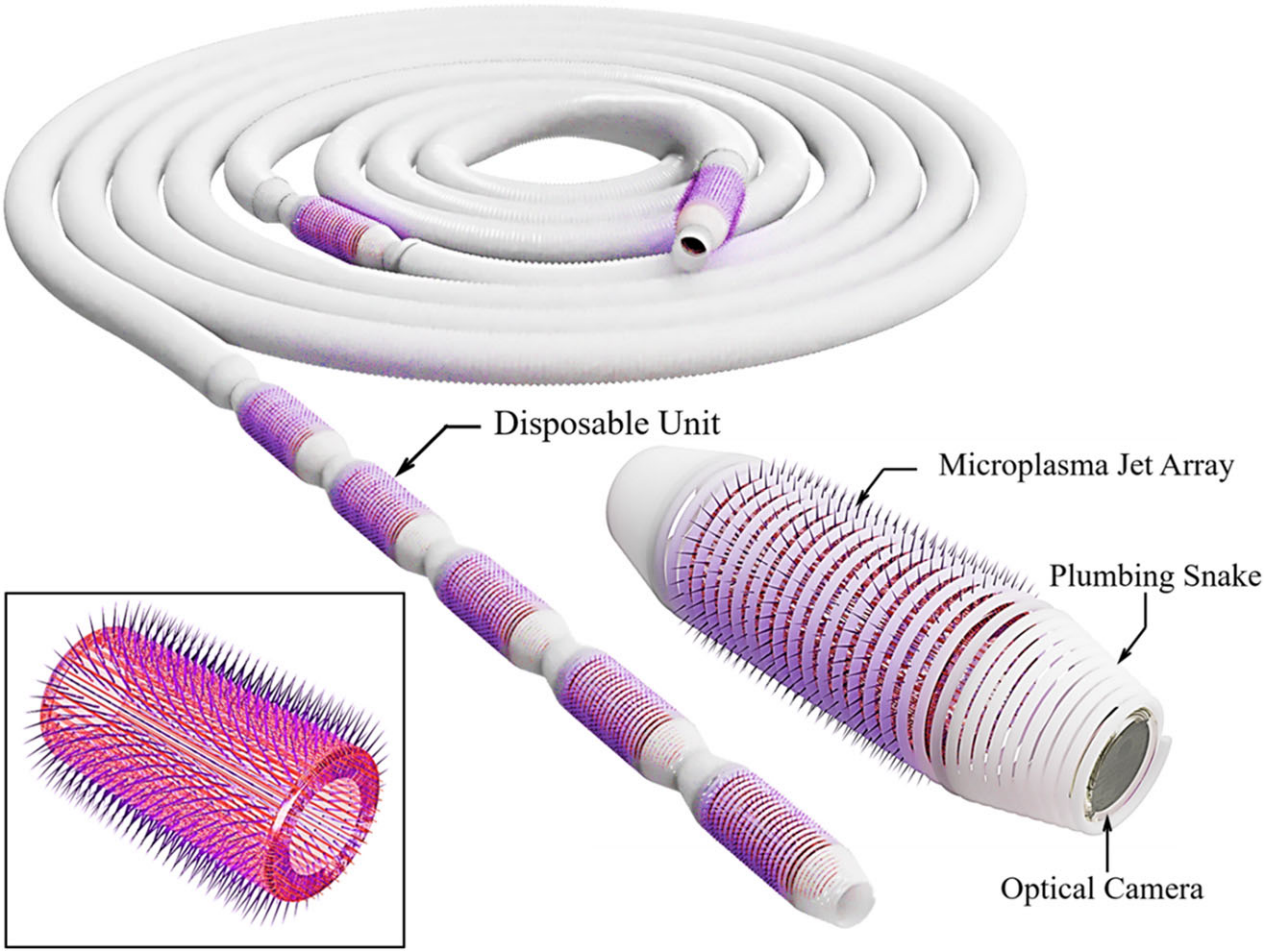
Illustration of a conventional plumbing snake modified so as to incorporate cylindrical sections generating arrays of radially—propagating plasma jets. A camera is installed at one end of the snake, and the inset is a qualitative drawing of one cylindrical microplasma array section (Ref.^22^)

Figure 6 presents several diagrams, reproduced from Ref.^22^, illustrating the structure of a biofilm treatment system comprising multiple cylindrical microplasma jet arrays integrated with a modified plumbing snake. The inset of Fig. 6 is a qualitative drawing (not to scale) of a cylindrical microjet array having a diameter sufficiently small so as to allow insertion of the array into the snake. Feedstock gas (which need not be helium) flows to each cylindrical array along the axis of the snake, which also accommodates electrical connections providing power to the jet arrays and a camera.

For the current electrical efficiency of the process described here, biofilm control or removal with microplasma jet arrays is economically viable. Assuming the complete removal of biofilm in 20 min. at an average power density of 60 W/cm^2^, the electrical power required is 0.02 kWh per cm^2^ of surface area (lower if complete biofilm removal is not necessary). Consequently, even if one assumes that engineering development of these arrays does not result in increased plasma generation efficiency and reduced treatment time, the cost of power associated with treating (2–10) × 10^5^ cm^2^ of surface area is not prohibitive. Because the plasma treatment of biofilms on the interior surface of water pipes necessarily confines the molecular radicals and excited species generated by the microplasmas, it is expected that biofilm removal rates in water distribution systems will be significantly larger than those reported here for the present (planar) geometry. As is evident in Fig. 3a, the present geometry allows radicals to escape along any plane transverse to the axes of the microplasma jets.

This technology appears to be of particular value for intervention in those circumstances in which residential or commercial plumbing is known to have been compromised by one or more pathogens, and previous and persistent efforts to purge or disinfect the system have proven unsuccessful. Arrays of microplasma jets provide a chlorine-free and *in situ* tool for controlling biofilms and pathogen populations with a degree of specificity (with regard to spatial localization and customized treatment of various biofilms) not available previously.

## Methods

### **Preparation of simulated drinking water biofilms**

Biofilms were grown on polyvinyl chloride (PVC) coupons from a groundwater source of drinking water in Urbana-Champaign, Illinois (USA). Treated by a greensand filter, this water was found to contain 1.65 ± 0.08 mM Ca^2+^, 1.16 ± 0.01 mM Mg^2+^, and 1.04 mM Na^+^, and its measured hardness is 281 ± 8 mg/L. The total organic content (TOC) of the groundwater is 1–1.6 mg/L, and the pH is in the range of 7.5–7.8. Because the process for growing biofilms has been described in detail previously^23,24^, it will be only briefly reviewed here. PVC coupons (RD 128-PVC, BioSurface Technologies Corporation, Bozeman, Montana) served as the substrate for the biofilms. After placing the coupons into CDC reactors (CBR 90-2, Biosurface Technologies Corporation), groundwater was pumped continuously into the reactors and biofilm development took place under shearing conditions because of continuous stirring within the reactors at 125 rpm, which corresponds to an Re value of 2384. No extraneous nutrients or microorganism strains were introduced to the reactors, and the biofilms were allowed to develop undisturbed for 10 months prior to the characterization and plasma treatment experiments.

### **Plasma treatment of biofilms**

All of the experiments reported here were conducted with the microplasma array of Fig. 1, driven by a 20 kHz sinusoidal waveform having an RMS value of 1.2 kV. Helium gas flow was maintained at 0.25 L/min/microchannel (approximately 20 l/min. total flow rate) and the exit face of the array was situated 4 mm from a PVC coupon onto which a biofilm had been grown. In total, seven biofilm coupons were examined in this study.

### **Biofilm imaging by optical coherence tomography, confocal laser scanning microscopy, scanning electron microscopy, and energy dispersive spectroscopy**

All biofilms were examined *in situ*, both prior to and after plasma treatment, by OCT^25,26^. A custom built, spectral domain OCT system, emitting low-coherence light with a central wavelength of 1322 nm and a bandwidth of 106 nm, offers an axial and transverse resolution of 4.2 µm and 3.9 µm, respectively, in air^27^. For each biofilm sample, cross-sectional images having a volume of 3.1 mm (transverse dimension) × 2.1 mm (depth) × 4 mm were recorded at three locations on the film, and 600 images were captured for each biofilm coupon. Seventy cross-sectional images were selected randomly for further analysis. Each of these images was processed by ImageJ software (http://imagej.nih.gov/ij/) so as to suppress or eliminate background noise. Also, the mean thickness of all biofilms was determined by analyzing the gray scale gradient through automatic thresholding with the MATLAB program developed by Derlov et al.^28^

After the completion of the OCT scans, the same biofilm samples were stained following the protocol of the LIVE/DEAD BacLight™ Bacterial Viability Kit (Thermo Fisher Scientific). This assay determines the viability of the cell membrane. Living cells were stained green with SYTO 9 while dead cells were stained red (propidium iodide) for subsequent evaluation by CLSM. Images were acquired with a Leica SP8 laser scanning confocal microscope in which photoexcitation occurs at 488 nm (Ar ion laser) and the detection wavelengths are 500 and 635 nm for SYTO 9 and propidium red, respectively. Final images were produced from the microscope data by LAS X software offered by Leica Microsystems, and red and green intensity maps were generated from the pixel data with ImageJ software and analysis by the MATLAB program COMSTAT.

As additional diagnostics of the microplasma treatment process, several biofilm coupons were also examined, after differing periods of microplasma exposure, by scanning electron microscopy (SEM) and energy dispersive spectroscopy (EDS).

### **Detection of microplasma-produced reactive oxygen species**

Knowledge of the absolute concentrations of several critical oxygen-bearing radicals and excited species, such as OH, ^1^O_2_, H_2_O_2_, and O_3_, is essential to assessing the efficacy of microplasmas for the deactivation of bacterial pathogens. To this end, tests were conducted in which a beaker 3.5 mm in height was situated immediately beneath the exit face of the microplasma arrays. The beaker contained a 10 mL solution of phosphate-buffered saline solution (PBS; pH = 7.4) and 100 µM of either phenol (99%, Acros Organics) or furfuryl alcohol (FFA; 98%, Acros Organics), the latter of which serve as a diagnostic of OH (phenol) or ^1^O_2_ (FFA), respectively. After the appropriate solution was exposed to the He plasma jets emerging into room air, samples were withdrawn and the decay of the desired probe was detected by liquid chromatography with an Agilent series 1200 HPLC chromatograph having an Eclipse Plus C18 (3.5 µm) column. Separation was performed with water and acetonitrile with a ratio of 50:50 for phenol and 40:60 for FFA, and the flow rate and injection volume were fixed at 0.3 mL/min and 20 µL, respectively. The detection wavelengths for phenol and FFA were 268 nm and 216 nm, respectively.

The concentration of the designated probe was calculated on the basis of the standard curve for the same probes in PBS^29^. For the OH radicals generated by the microplasma array, the concentration in the beaker solution was determined from the decay of phenol in solution and is given by the product of the rate constant for the phenol-OH interaction (1.4 × 10^10^ M^−1^ s^−1^)^30^, and the phenol concentration. Similarly, the ^1^O_2_ concentration was determined from the product of the ^1^O_2_—FFA interaction rate constant (1.4 × 10^8^ M^−1^s^−1^)^23,31^, and the FFA concentration. Measurements of the hydrogen peroxide concentration were facilitated by a colorimetric test kit (Hydrogen Peroxide—CHEMets® Visual Kit). Hydrogen peroxide oxidizes ferrous iron to the ferric state, resulting in the formation of a red thiocyanate complex. By comparing the colors of the test samples with those of standard samples (provided by the test kit), the concentrations of H_2_O_2_ in the test samples were determined. The minimum detectable value was 1.5 µM. Finally, the concentration of ozone dissolved in solution was measured with a second test kit (HACH OZ-2 (2064400)). Each test was performed at least three times, in order to obtain a statistically significant result.

## Electronic supplementary material

Supplementary Information

## Data Availability

All data supporting the findings of this study are available from the corresponding author upon request.

## Electronic supplementary material

**Supplementary information** accompanies the paper on the *npj Biofilms and Microbiomes* website (10.1038/s41522-018-0063-4).

## References

[1] Lau H.Y., Ashbolt N.J. (2009). The role of biofilms and protozoa inLegionellapathogenesis: implications for drinking water. Journal of Applied Microbiology.

[2] Tison, D. L., Pope, I. D. H., Cherry, W. B. & Fliermans, C. B. Growth of *Legionella pneumophila* in association with blue- green algae (Cyanobacteria). *Appl. Environ. Microbiol.* **39**, 456–459 (1980). 10.1128/aem.39.2.456-459.1980 PMC291353 6769388

[3] Wadowsky, R. M. & Yee, R. B. Satellite growth of *Legionella**pneumophila* with an environmental isolate of *Flavobacterium breve*. *Appl. Environ. Microbiol.* **46**, 1447–1449 (1983). 10.1128/aem.46.6.1447-1449.1983 PMC239593 6660882

[4] Stout, J. E., Yu, V. L. & Best, M. G. Ecology of *Legionella**pneumophila* within water distribution systems. *Appl. Environ. Microbiol.* **49**, 221–228 (1985). 10.1128/aem.49.1.221-228.1985 PMC238374 3977311

[5] Temmerman R., Vervaeren H., Noseda B., Boon N., Verstraete W. (2006). Necrotrophic Growth of Legionella pneumophila. Applied and Environmental Microbiology.

[6] Thomas JM, Ashbolt NJ (2011). Do free-living amoebae in treated drinking water systems present an emerging health risk?. Environ. Sci. Technol..

[7] Cargill Kari L., Pyle Barry H., Sauer Richard L., McFeters Gordon A. (1992). Effects of culture conditions and biofilm formation on the iodine susceptibility of Legionella pneumophila. Canadian Journal of Microbiology.

[8] Cooper I.R., Hanlon G.W. (2010). Resistance of Legionella pneumophila serotype 1 biofilms to chlorine-based disinfection. Journal of Hospital Infection.

[9] Kotay S, Chai W, Guilford W, Barry K, Mathers AJ (2017). Spread from the sink to the patient: *in situ* study using green fluorescent protein (GFP) – expressing *Escherichia coli* to model bacterial dispersion from–washing sink–trap reservoirs. Appl. Environ. Microbiol..

[10] Kim B.R., Anderson J.E., Mueller S.A., Gaines W.A., Kendall A.M. (2002). Literature review—efficacy of various disinfectants against Legionella in water systems. Water Research.

[11] Bridier A, Briandet R, Thomas V, Dubois-Brissonnet F (2011). Resistance of bacterial biofilms to disinfectants: a review. Biofouling.

[12] Becker KH, Schoenbach KH, Eden JG (2006). Microplasmas and applications. J. Phys. D. Appl. Phys..

[13] Graves DB (2012). The emerging role of reactive oxygen and nitrogen species in redox biology and some implications for plasma applications to medicine and biology. J. Phys. D. Appl. Phys..

[14] Pan Jie, Sun Ke, Liang Yongdong, Sun Peng, Yang Xiaohui, Wang Jing, Zhang Jue, Zhu Weidong, Fang Jing, Becker Kurt H. (2013). Cold Plasma Therapy of a Tooth Root Canal Infected with Enterococcus faecalis Biofilms In Vitro. Journal of Endodontics.

[15] Li Yinglong, Sun Ke, Ye Guopin, Liang Yongdong, Pan Hong, Wang Guomin, Zhao Yijiao, Pan Jie, Zhang Jue, Fang Jing (2015). Evaluation of Cold Plasma Treatment and Safety in Disinfecting 3-week Root Canal Enterococcus faecalis Biofilm In Vitro. Journal of Endodontics.

[16] Sun Yi, Yu Shuang, Sun Peng, Wu Haiyan, Zhu Weidong, Liu Wei, Zhang Jue, Fang Jing, Li Ruoyu (2012). Inactivation of Candida Biofilms by Non-Thermal Plasma and Its Enhancement for Fungistatic Effect of Antifungal Drugs. PLoS ONE.

[17] Sun P (2011). Atmospheric pressure cold plasma as an antifungal therapy. Appl. Phys. Lett..

[18] Xu Zimu, Shen Jie, Zhang Zelong, Ma Jie, Ma Ronghua, Zhao Ying, Sun Qiang, Qian Shulou, Zhang Hao, Ding Lili, Cheng Cheng, Chu Paul K., Xia Weidong (2015). Inactivation Effects of Non-Thermal Atmospheric-Pressure Helium Plasma Jet onStaphylococcus aureusBiofilms. Plasma Processes and Polymers.

[19] Alshraiedeh Nida H., Higginbotham Sarah, Flynn Padrig B., Alkawareek Mahmoud Y., Tunney Michael M., Gorman Sean P., Graham William G., Gilmore Brendan F. (2016). Eradication and phenotypic tolerance of Burkholderia cenocepacia biofilms exposed to atmospheric pressure non-thermal plasma. International Journal of Antimicrobial Agents.

[20] Taghizadeh L (2014). Inactivation of biofilms using a low power atmospheric pressure argon plasma jet; the role of entrained nitrogen. Plasma Process. Polym..

[21] Sun PP, Cho JH, Park CH, Park SJ, Eden JG (2012). Close-packed arrays of plasma jets emanating from microchannels in a transparent polymer. IEEE Trans. Plasma Sci..

[22] Eden, J. G. & Sun, P. P. Microplasma devices for surface or object treatment and biofilm removal, U.S. Patent Application No. 15/98105 (2018).

[23] Janjaroen Dao, Ling Fangqiong, Monroy Guillermo, Derlon Nicolas, Mogenroth Eberhard, Boppart Stephen A., Liu Wen-Tso, Nguyen Thanh H. (2013). Roles of ionic strength and biofilm roughness on adhesion kinetics of Escherichia coli onto groundwater biofilm grown on PVC surfaces. Water Research.

[24] Shen Yun, Huang Conghui, Monroy Guillermo L., Janjaroen Dao, Derlon Nicolas, Lin Jie, Espinosa-Marzal Rosa, Morgenroth Eberhard, Boppart Stephen A., Ashbolt Nicholas J., Liu Wen-Tso, Nguyen Thanh H. (2016). Response of Simulated Drinking Water Biofilm Mechanical and Structural Properties to Long-Term Disinfectant Exposure. Environmental Science & Technology.

[25] Xi C, Marks D, Schlachter S, Luo W, Boppart SA (2006). High-resolution three-dimensional imaging of biofilm development using optical coherence tomography. J. Biomed. Opt..

[26] Shen Yun, Monroy Guillermo L., Derlon Nicolas, Janjaroen Dao, Huang Conghui, Morgenroth Eberhard, Boppart Stephen A., Ashbolt Nicholas J., Liu Wen-Tso, Nguyen Thanh H. (2015). Role of Biofilm Roughness and Hydrodynamic Conditions in Legionella pneumophila Adhesion to and Detachment from Simulated Drinking Water Biofilms. Environmental Science & Technology.

[27] Ahmad Adeel, Shemonski Nathan D., Adie Steven G., Kim Hee-Seok, Hwu Wen-Mei W., Carney P. Scott, Boppart Stephen A. (2013). Real-time in vivo computed optical interferometric tomography. Nature Photonics.

[28] Derlon N, Peter-Varbanets M, Scheidegger A, Pronk W, Morgenroth E (2012). Predation influences the structure of biofilm developed on ultrafiltration membranes. Water Res..

[29] Rosado-Lausell SL (2013). Roles of singlet oxygen and triplet excited state of dissolved organic matter formed by different organic matters in bacteriophage MS2 inactivation. Water Res..

[30] Alexieva Zlatka, Yemendzhiev Hyusein, Zlateva Plamena (2010). Cresols utilization by Trametes versicolor and substrate interactions in the mixture with phenol. Biodegradation.

[31] Haag Werner R., Hoigne Juerg. (1986). Singlet oxygen in surface waters. 3. Photochemical formation and steady-state concentrations in various types of waters. Environmental Science & Technology.

