# Supplementary material for: Disintegration of simulated drinking water biofilms with arrays of microchannel plasma jets
Source: NPJ Biofilms Microbiomes. 2018 Oct 18;4:24. doi: 10.1038/s41522-018-0063-4 (PMC6194111; doi:10.1038/s41522-018-0063-4)
Supplement: Supplementary file 1 — Supplementary Information [file 41522_2018_63_MOESM1_ESM.docx]

SUPPLEMENTARY FIGURE CAPTIONS

Fig. 1S


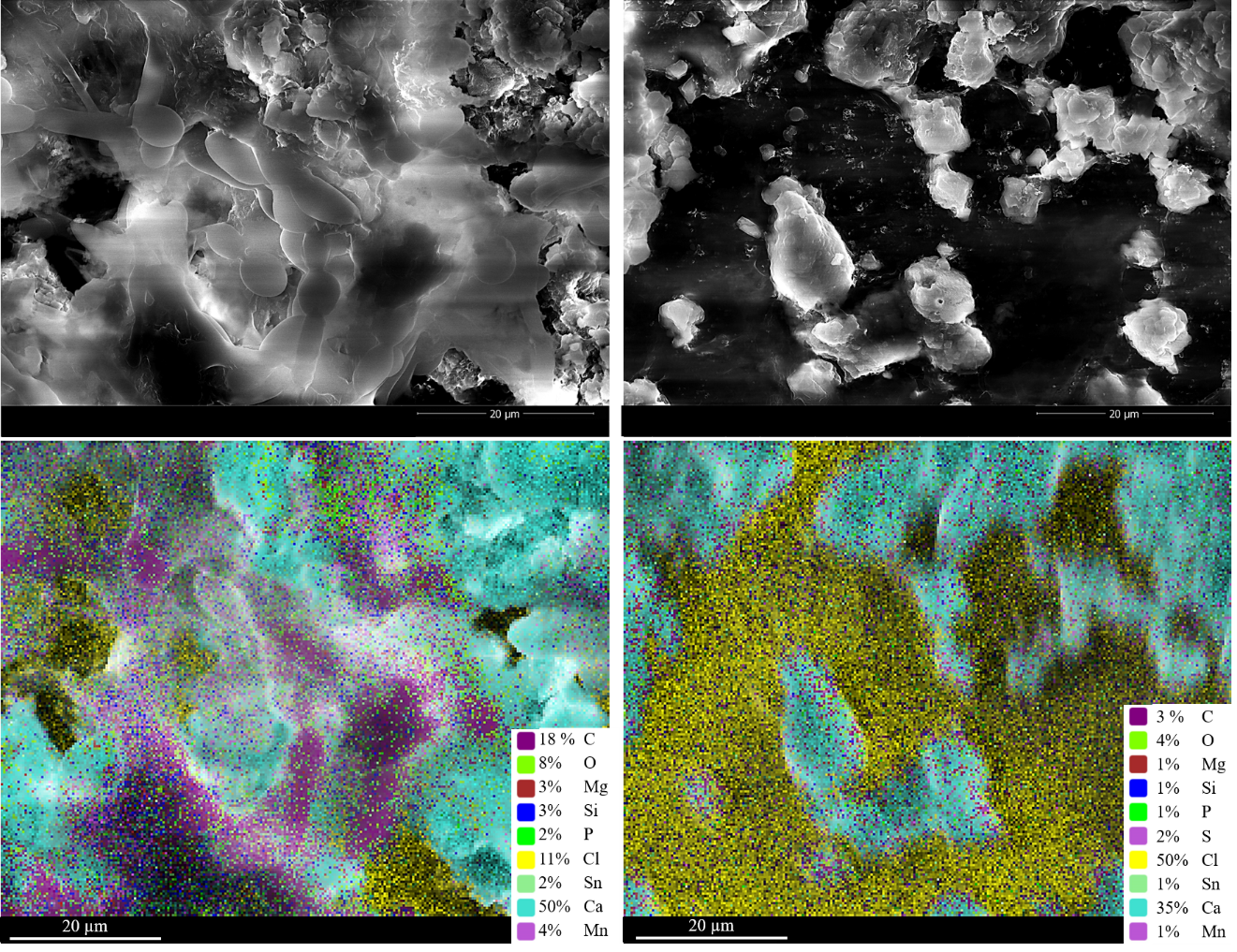


Fig. 1S Images of SEM and EDS of the groundwater biofilm before and after microplasma jet array treatment.

Fig. 2S


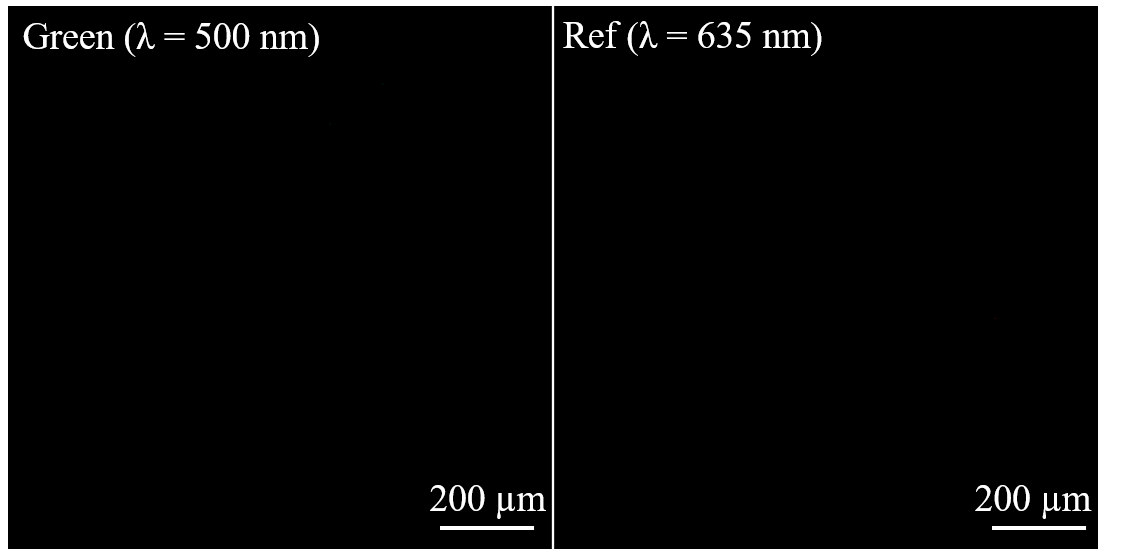


Fig. 2S Confocal laser scanning microscopy images recorded of the dispersed biofilm fragments, when staining with SYTO 9 (green, λ = 500 nm), or propidium red (λ= 635 nm). No fluorescence signal was detected in either of the two channels.

Fig. 3S


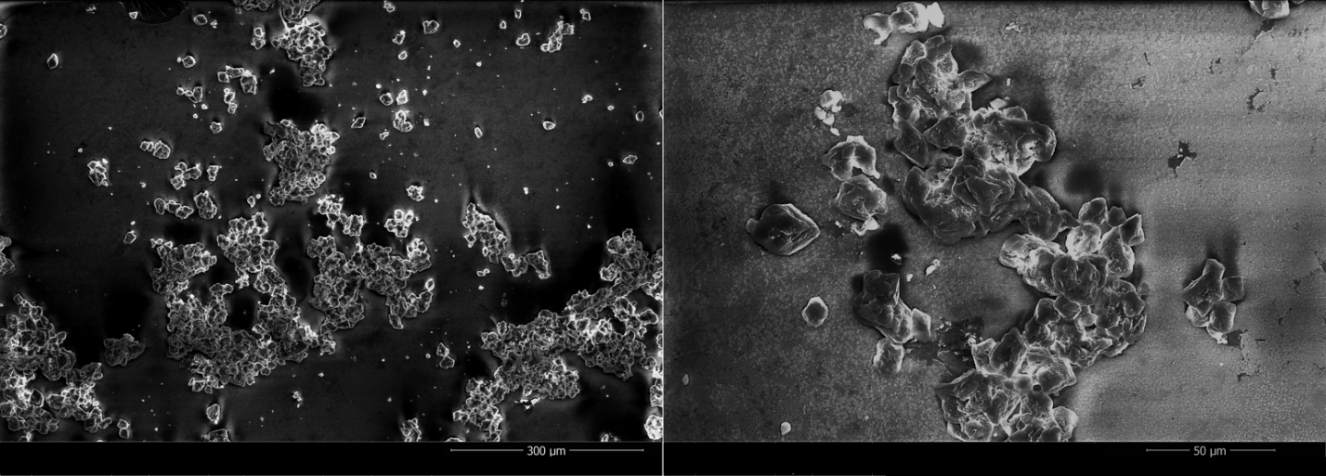


Fig. 3S SEM images of dispersed biofilm fragments produced by microplasma jet array treatment. The dispersed biofilm was collected and centrifuged prior to electron microscopy analysis.

Fig 4S

Fig 4S Temporal history of phenol decomposition by the microplasma jet array in the absence or presence of the OH scavenger mannitol.
